# The Role of the Coat Protein A-Domain in P22 Bacteriophage Maturation

**DOI:** 10.3390/v6072708

**Published:** 2014-07-14

**Authors:** David S. Morris, Peter E. Prevelige

**Affiliations:** Department of Microbiology, University of Alabama at Birmingham, 845 19th Street S, BBRB 414, Birmingham, AL 35294, USA; E-Mail: davidsm@uab.edu

**Keywords:** procapsid, bacteriophage, maturation, recombineering

## Abstract

Bacteriophage P22 has long been considered a hallmark model for virus assembly and maturation. Repurposing of P22 and other similar virus structures for nanotechnology and nanomedicine has reinvigorated the need to further understand the protein-protein interactions that allow for the assembly, as well as the conformational shifts required for maturation. In this work, gp5, the major coat structural protein of P22, has been manipulated in order to examine the mutational effects on procapsid stability and maturation. Insertions to the P22 coat protein A-domain, while widely permissive of procapsid assembly, destabilize the interactions necessary for virus maturation and potentially allow for the tunable adjustment of procapsid stability. Future manipulation of this region of the coat protein subunit can potentially be used to alter the stability of the capsid for controllable disassembly.

## 1. Introduction

**Figure 1:**
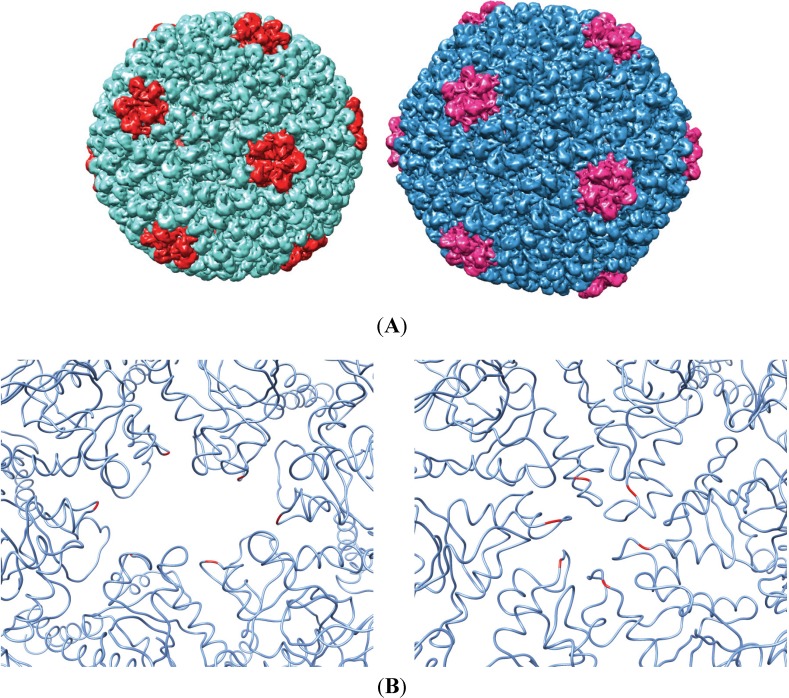
(**A**) Depiction of an immature (**left**) and mature (**right**) coat protein shell. Pentameric capsomeres are highlighted for reference. (**B**) Hexameric capsomeres of the immature (**left**) and the mature structure with the surface res1and the A-domain residue T183 highlighted. Upon maturation, the expansion of the virus results in a conformational shift that restricts the size of the pore in the middle of each capsomere (see Movie S1). The figures were generated from Protein Data Bank (PDB) 2XYZ and 2XYY using the UCSF Chimera package [12,13].

Bacteriophage P22 is a dsDNA virion of the *Podoviridae* family that infects *Salmonella enterica* serovar *typhimurium*. In the native host, co-expression of the major structural proteins, coat, portal and scaffolding (gp5, gp1 and gp8, respectively), among other minor proteins, results in the assembly of a T = 7 L icosahedral quasi-equivalent procapsid structure 50 nm in diameter [1,2,3]. Packaging of the phage DNA into the procapsid results in the exit of scaffolding protein and the subsequent expansion of the capsid shell (Figure 1A). This maturation process requires significant changes in the intersubunit contacts of the phage coat protein, including the closing off of a “pore” in the center of each coat protein capsomere (Figure 1B). Recently, phage procapsids have been utilized for a number of alternative purposes, including biomedical applications, such as nanoparticle targeted drug delivery [4,5,6]. To make P22, an effective vector for nanomedicine, we were interested in developing a phage display system on the surface of the P22 capsid by manipulating sequences of the P22 coat protein *in vivo*. The A-domain of the coat protein was chosen for these manipulations, because previous work has described this loop as flexible and solvent exposed, and manipulations of this region at residue T183 *in vitro* were highly permissive of procapsid assembly [7,8]. Residue T183 was chosen in these studies, because models of the P22 procapsid structure place this residue at the most axial position in each capsomere. While several A-domain peptide insertion sequences had been examined *in vitro*, the focus initially was on the tri-peptide sequence: RGD. The RGD sequence (Arg-Gly-Asp) interacts preferentially with the α_V_β_3_ integrin, a common cell surface receptor [9,10]. While α_V_β_3_ integrin is expressed on many cell surfaces, it is highly upregulated in cancerous cells and has been examined as a potential target for the drug delivery of toxic chemotherapies. As a proof of concept for developing the phage display library, the RGD sequence flanked by two tri-glycine linkers (GGGRGDGGG) was inserted at residue T183 into the A-domain of P22 coat protein, and the resulting procapsids were thoroughly characterized, both *in vitro* through a BL21 expression system that produces non-infectious P22 procapsids, as well as *in vivo* utilizing a stable lysogen of P22 and lambda-red recombineering. Upon finding that the RGD sequence allowed for assembly, but inhibited virus maturation, insertions of decreasing complexity were made in the A-domain in order to determine the sequence permissiveness of the region. Our results show how the manipulation of the coat protein *in vivo* can be used to study the biophysical properties of virus maturation. The insight gained from this work indicates that structural changes in the A-domain of the P22 coat protein can controllably manipulate the ability of the phage to mature, while allowing for procapsid assembly.

## 2. Materials and Methods

### 2.1. Producing P22 Procapsids

BL21 cells containing the fluorescent P22 assembler plasmids (GFP/131-303gp8-5 or mCHERRY/131-303gp8-5 contained within a pET11b vector) were grown to 0.6 OD under antibiotic selection [11]. Protein expression was induced by adding 1 mM IPTG to the culture and allowing for an additional 3.5–4 h incubation at 37 °C while shaking. Following expression, procapsids were purified as previously described. Pelleted cells were subjected to three freeze/thaw cycles and then lysed by sonication. Cell debris was cleared through centrifugation at 13,000 RPM for 1 h in a JA-20 rotor. Procapsids in solution were then pelleted through 20% sucrose at high speed (40 K 2 h 70Ti rotor) and re-suspended in Buffer B (50 mM Tris, 25 mM NaCl, 2 mM EDTA) prior to being subjected to an isopynic 5%–20% sucrose gradient designed to separate out procapsids from aggregated material (35 K, 35 min, SW55 rotor). The procapsid band was then isolated and dialyzed against Buffer B for short-term storage. The P22 assembler plasmids were mutated using the Quickchange site-directed mutagenesis method. The primers used are described in Table S1.

### 2.2. Heat Expansion Assay

Isolated procapsids were buffer exchanged to 50 mM NaP0_4_, 1 mM MgCl_2_, pH 7.4, and the concentration of gp5 was standardized to 20 mM by arithmetically removing the contribution to absorbance generated by the fluorescent scaffolding fusion. Fifty-microliter aliquots of the sample were heated at the times and temperatures described using thin-walled PCR tubes in a Bio-Rad iCycler themocycler held at a constant temperature. Post-heated samples were cooled to 4 °C and then run on a 1% agarose gel at 50 V for 1 h. Gels were stained overnight with Coomassie stain followed by destaining with a 10% acetic acid, 30% methanol solution until thoroughly destained. The quantification of bands was performed using ImageJ software by plotting intensities for each lane and integrating under each curve after baseline subtraction [14].

### 2.3. Recombineering

*S. enterica* serovar *typhimurium* LT2 strains containing stable P22 lysogens were obtained from Sherwood Casjens. UB-1757 (leuA–414, ΔFels2, r-, sup° containing P22 15–ΔSC302::KanR 13–amH101) and UB-2158 (leuA-414, ΔFels2, r-, sup°, ΔGalK:TetRA containing P22 c1-7, 13-amH101, ΔsieA-1, orf25::CamR-EG1) were used. Lamda-red recombineering [15,16] was performed on P22 gene 5 using the primers described in the supplementary figures. Either the TetRA tetracycline resistance cassette (Tn10dTc) or the galK galactose kinase cassette (from pgalK) was PCR amplified using primers with 3’ end homology to the cassette and 40 bp 5’ homology to the gp5 sequences specific to the A-domain (TetRP22for/rev or galKP22for/rev). The resulting amplicon was then PCR purified and electroporated into UB-1757 or UB-2158 electrocompetent cells, containing an arabinose induced PKD46 plasmid. Cells were then selected for tetracycline resistance or galK activity, and resulting colonies were tested by PCR and sequencing. Mutant sequences with flanking homology to the target gene were PCR amplified using the oligo extension technique, and subsequent lambda-red recombineering into the TetRA or galK interrupted strain allows for the restoration of the gene coding sequence with the mutation of interest. Selection for tetracycline sensitive strains was done twice on Bochner-Maloy media and reconfirmed by stippling onto tetracycline and kanamycin agar plates. Selection for ΔgalK strains was performed on M63 minimal media agar in the presence of glucose and 2-deoxy-galactose (2-DOG) as previously described [16].

### 2.4. Phage Induction and Titering

Induction of the lysogen containing *S. typhimurium* strains was performed by growing cells to 0.6 OD in LB followed by at least two hours of induction with carbodox (1 μg/mL) or mitomycin C (0.5 μg/mL) at 37 °C, unless mentioned otherwise. Cells were pelleted and incubated with a saturating volume of chloroform to complete lysis and release of infectious phage. Cell debris was pelleted by centrifugation, and the phage containing supernatant was isolated. Tailspike protein is poorly produced in the UB-1757 P22 lysogen, which required the addition of saturating amounts of the exogenously produced tailspike to get accurate titers. Phages were titered on the MS-1363 suppressor strain (leuA–am414 supE) on soft agar containing 10 mM citrate in order to assist with cell lysis. Supplementation of exogenous tailspike and plating in the presence of 10 mM citrate were not necessary for phage produced from the UB-2158 strain. Phage product was concentrated by centrifugation and sedimented through a 5%–20% sucrose gradient using the same methods as shown above for procapsids. Imaging of the fractions was performed by negative stain TEM (2% uranyl acetate) after dialysis into Buffer B.

## 3. Results

### 3.1. In Vitro Maturation by Heat Expansion

**Figure 2:**
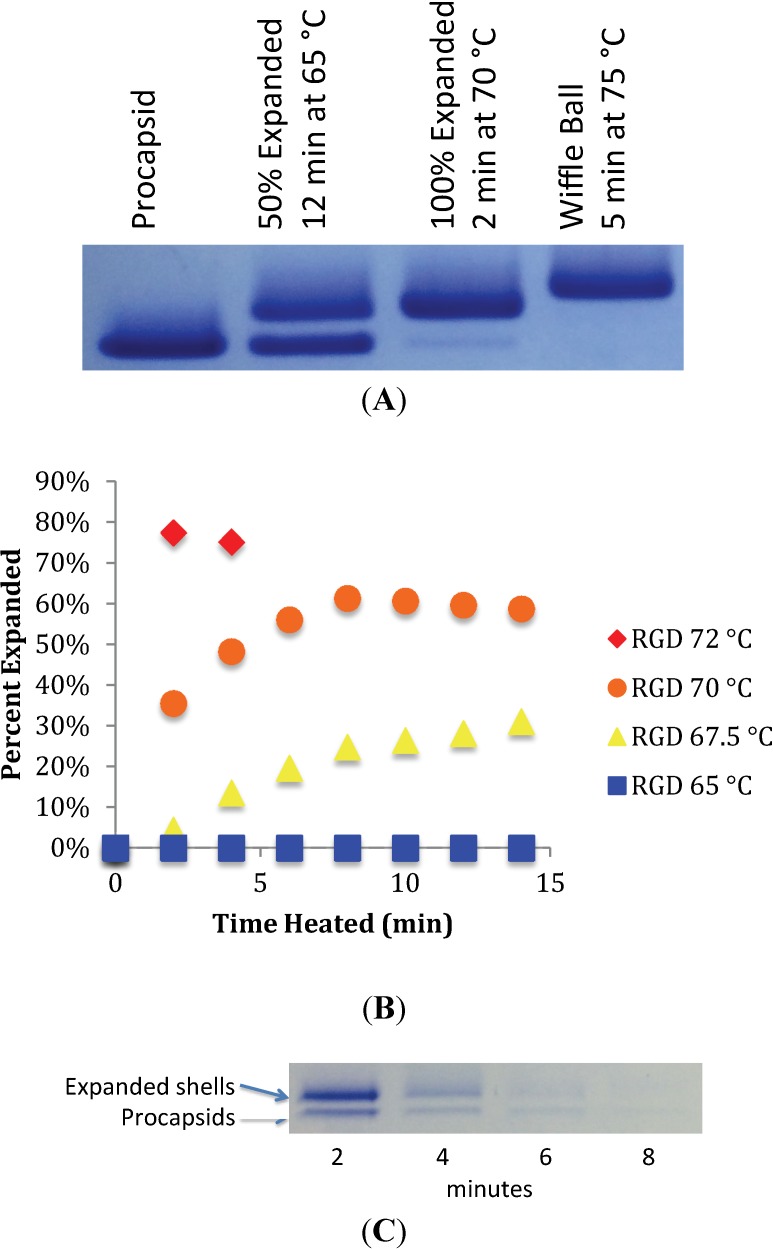
(**A**) Heat expansion of wild-type procapsids *in vitro*. Three forms can be distinguished based on migration on native agarose gel. Immature procapsids migrate the fastest, followed by expanded shells and, finally, by the “wiffle ball” form. (**B**) RGD procapsids (T183GGGRGDGGG) were heat expanded at constant temperature and electrophoresed on native agarose. Bands associated with the migration of expanded capsids and unexpanded procapsids were quantified by densitometry using ImageJ, and the relative values were plotted. (**C**) Heating RGD procapsids at 72 °C results in a reduction of overall staining intensity, suggesting shell dissociation. The “wiffle ball” morphology is not observed.

Heat expansion of P22 procapsids has been used previously to mimic the process of maturation *in vitro* [17]. Incubation of procapsids for 12 min at 65 °C produces expanded shells. Native gel electrophoresis can readily resolve unexpanded, expanded and wiffle ball forms. To characterize the ability of mutant RGD procapsids to mature *in vitro*, heat expansion followed by native agarose gel electrophoresis was performed. Shells standardized to 20 μM of P22 coat protein were heated in thin-walled PCR tubes at constant temperature and analyzed by migration on a 1% native agarose gel. Wild-type procapsids containing scaffolding protein behaved in a similar manner to previously published results [17] (Figure 2A).

Heat expansion at 65 °C for 12 min results in approximately 50% expansion, while at 70 °C, full expansion occurs within 2 min. Heating at 75 °C results in the formation of the wiffle ball form, resulting in the loss of coat protein capsomeres at each of the five-fold vertices of the icosahedral T = 7 shell. In contrast, we did not observe expansion of the RGD procapsids when heated at 65 °C, even when heating was continued for 15 min (Figure 2B). To further define the kinetics of expansion and to determine the relative stability of the capsid, heat expansion was performed over a range of temperatures. The transition to the expanded form became apparent when RGD procapsids were heated at 67.5 °C and 70 °C. However, even at 70 °C, the transition was slower than was observed for the wild-type indicating, that the RGD insertion to the A-domain loop increases the activation energy required to transition to the expanded form. When heating at 72 °C or higher, the RGD procapsids were destabilized and dissociated completely (Figure 2C). There were no conditions at which the “wiffle ball” form of the RGD procapsid was observed. Taken together, these observations demonstrate that the RGD insertion at the A-domain loop both increases the energy required to transition to the mature-like morphology, while also destabilizing this form of the capsid.

### 3.2. Testing the A-Loop Insertion in Vivo

**Figure 3:**
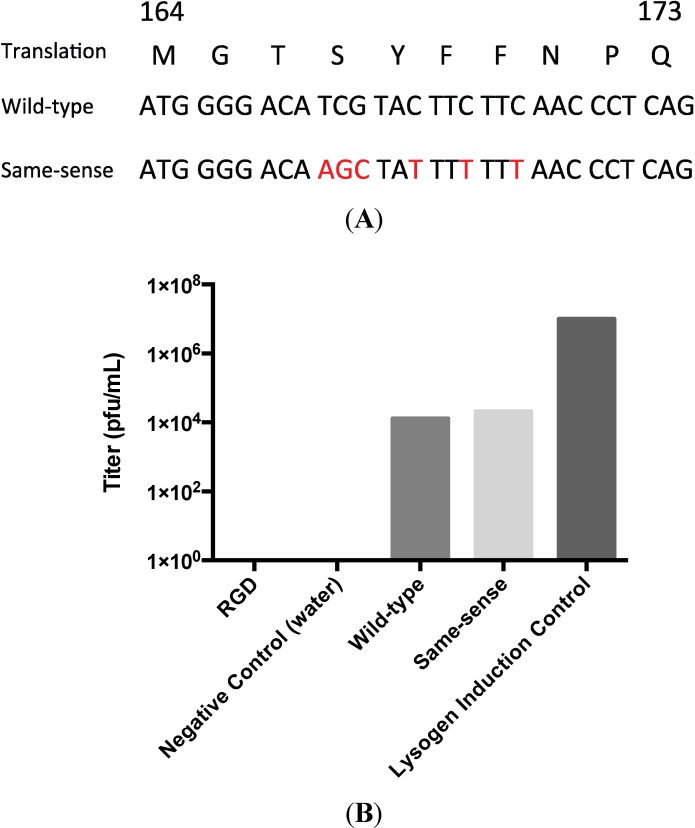
(**A**) The custom-designed same-sense nucleotide sequence as compared with the wild-type. Red nucleotides are different from wild-type without changing the amino acid coding. (**B**) Phage titer results post-recombineering indicated that recombineering was successful due to the presence of titer for both the wild-type and same-sense reactions. RGD does not produce infectious phage. Titers are representative of multiple recombineering reactions.

With the goal of utilizing the A-domain loop as a site for random insertions to develop a P22 phage display system, we inserted a tetracycline resistance cassette (tetRA) at residue T183 of the A-domain of the P22 lysogen by recombineering. Verification of the correct positioning of the cassette was performed by PCR using strategically-placed primers and determining the amplicon size (Figure S1). As expected, the induction of this lysogen did not result in the production of infectious phage. Amplicons encoding both the RGD sequence, the wild-type sequence and an alternatively-coded wild-type sequence (termed same-sense) were generated (Figure 3A) and recombineered back into the tetRA disrupted lysogen. The population of cells was induced, and the resultant phage titered (Figure 3B).

Both the wild-type and same-sense reactions produced phage with an efficiency of approximately three orders of magnitude less than the undisrupted lysogen. This presumably reflects the efficiency of the recombineering reaction. Sequencing of the isolated same-sense phage confirmed that the infectious phenotype was due to the insertion of the PCR product into the coding sequence. In contrast, no viable phage were detected in the RGD recombinant. To insure that recombination had occurred, counter-selection for tetracycline sensitivity using Bochner-Maloy plates was performed to isolate clonal populations of *S. typhimurium* [15,18,19]. Sequencing confirmed that a clonal population of tetracycline-sensitive cells harbored the RGD lysogen. A recombineering-based back-cross in which the wild-type sequence was reintroduced into the RGD lysogen produced infectious phage, confirming that the only mutation blocking infectivity was the RGD insertion. Thus, while the RGD insertion does not appear to block procapsid-like particle assembly in an expression system and those particles are capable of undergoing heat-induced expansion, the RGD lysogen is incompatible with the production of infectious phage.

### 3.3. Biochemical Characterization of the RGD Lysogen

**Figure 4:**
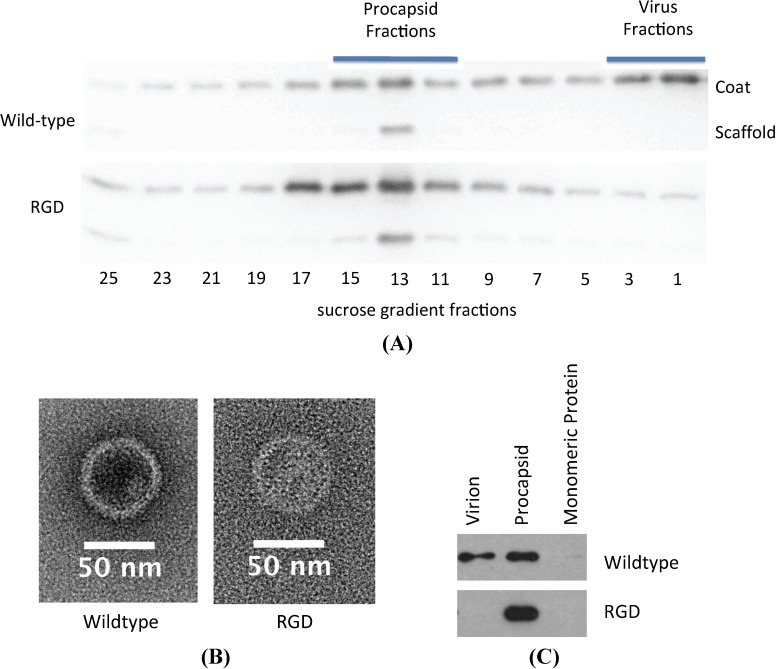
(**A**) Sucrose gradient fractionation of procapsid and mature morphologies indicates that the RGD strain only produces procapsids. (**B**) TEM of particles isolated from the procapsid fraction of the sucrose gradient confirm the similar morphology between wild-type and RGD morphologies; 2% uranyl acetate. (**C**) Western blot probing for P22 portal protein shows that RGD procapsids have portal protein present.

The absence of infectious phage particles could be caused by any number of failures in the assembly and maturation pathway of P22. Procapsid assembly was assayed to determine if the RGD insertion was preventing folding or assembly of the coat protein subunits. Strains of *S. typhimurium* containing both the wild-type and RGD lysogens were induced and the products analyzed by sedimentation of the lysates through a sucrose gradient. Western blots of the resulting fractions demonstrated peaks of coat and scaffolding protein co-sedimenting to a position typical of procapsids in both samples and a peak of coat protein in the phage position only for the wild-type sample (Figure 4A). Examination of the corresponding procapsid and virion fractions by TEM demonstrate that both the RGD and wild-type procapsids have a similar morphology (Figure 4B). Mature phage particles were not observed in the pellet fraction of RGD, whereas they were observed frequently in the corresponding wild-type fraction. This data suggests that RGD containing coat protein can co-assemble with scaffolding protein into procapsids, but not mature into phage. Incorporation of the portal dodecamer complex into the assembled procapsid is required for the procapsid to be capable of packaging DNA [20]. To determine whether or not portal was incorporated into the RGD procapsids, the sucrose gradient fractions were probed for the presence of portal (Figure 4C). As expected, in the case of the wild-type gradient, portal protein was detected in both the virion and the procapsid forms. Portal protein was also detected in the RGD procapsid, effectively proving that portal can be successfully assembled into RGD versions of the coat protein. The escape of scaffolding protein from the procapsid is required for DNA packaging. To gauge if the scaffolding exit was playing a role in the blocking maturation, scaffold escape was mimicked *in vitro* using guanidine hydrochloride. Treatment with guanidine hydrochloride indicated that the scaffolding protein contained within the RGD procapsid-like particle is retained more robustly than in the wild-type (Figure S2).

### 3.4. Additional Manipulations of the A-Domain

A series of additional insertions designed to discriminate between sequence specific and steric effects were recombineered into the tetRA interrupted lysogen (Table 1).

**Table 1:** Mutations of P22 coat generated by lambda-red recombineering.

Mutations Generated in 1757/TetRA	Mutations Generated in 2158/galK
T183GGGRGDGGG	T183AAAA
T183GGGGGGGGG	T183AAA
T183RGD	T183AA
T183GGG	T183A

**Figure 5:**
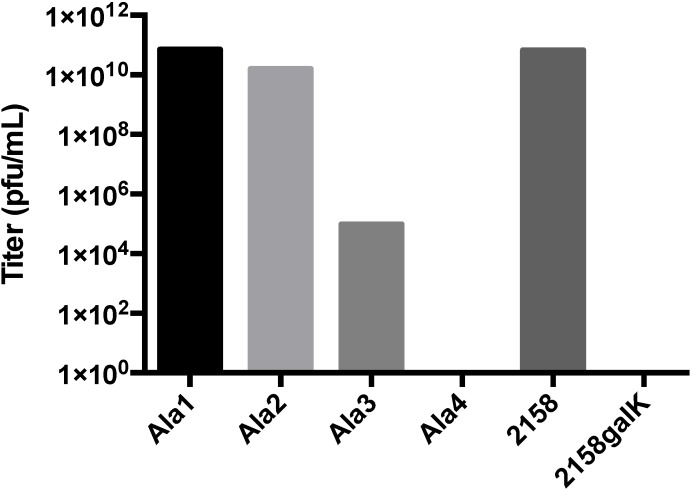
Induction and titer of phages produced from clonal mutants. 2158 is the original strain containing the wild-type lysogen. 2158 galK is the background strain for recombineering that contains a galK cassette within the coat gene. Ala1-4 are the single-, double-, triple- and quadruple-alanine insertions generated by lambda-red recombineering. Results are representative of multiple induction preparations.

**Figure 6:**
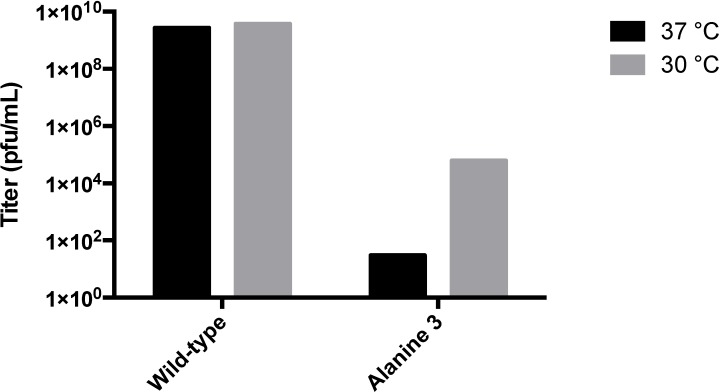
Temperature sensitivity test. Strains containing either wild-type or triple-alanine lysogens were induced at 30 °C for three hours and titered on DB-1636 at 37 °C or 30 °C. No significant differences were observed at either temperature for the wild-type phage. Triple-alanine insertion mutants exhibited a large increase in titer, confirming the temperature-sensitive phenotype. Relative titer results are representative of multiple inductions.

**Figure 7:**
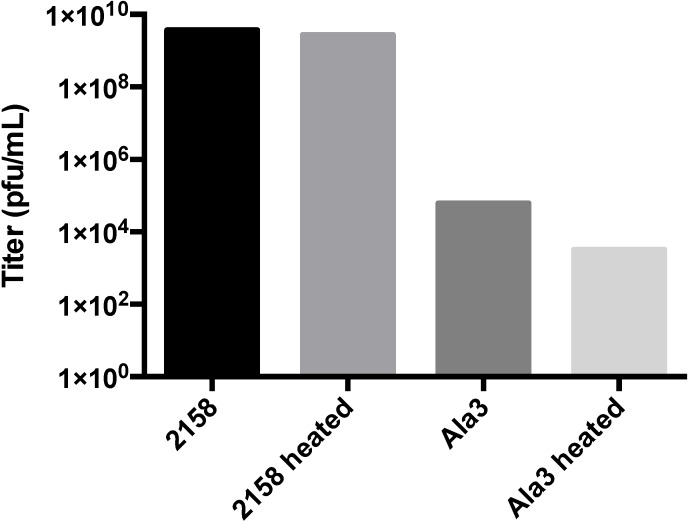
Heat stability test. Induced phage from 30 °C induction were isolated and incubated at room temperature or heated to 37 °C for 2 h. Titer incubation was performed at the permissive temperature, 30 °C. Heating the mutant phage results in a greater than 1.2 log reduction in the overall phage titer. Relative titer results are representative of multiple inductions.

In all cases, clonal populations of cells with the interrupted lysogen were isolated and confirmed by sequencing. Replacing the glycine flanked RGD sequence (T183GGGRGDGGG) with nine glycine residues did not produce infectious particles, suggesting that steric factors were paramount. Insertion of unflanked RGD (T183RGD) or T183GGG similarly abolished infectious phage production (Figure S3). This data strongly suggests that the blocking of phage production was most likely a steric hindrance effect on maturation. To further define the extent to which steric hindrance plays a role in the failed maturation of A-domain manipulated phage, we generated alanine insertions of decreasing length (T183AAAA, T183AAA, T183AA and T183A) at the A-domain loop. Clonal strains of *S. typhimurium* containing each of the four alanine mutant lysogenic P22 genomes were isolated and induced to produce phage (Figure 5). While both the single- and double-alanine insertions were capable of producing near wild-type phage titers, the triple alanine insertion resulted in a significant drop in titer, and the quadruple insertion was generally unable to produce plaques or did so at near detection limit levels (<10 pfu/mL). Plaques obtained from the alanine mutants were sequenced to determine the genotype of the resulting phage. The wild-type, single- and double-alanine mutants all contained the respective nucleotide sequences. Sequencing of plaques obtained from the triple and quadruple insertion mutants indicated that the genotype of these rare plaques were actually revertant double‑alanine insertions, suggesting a strong selection pressure against insertions of three residues or greater. In addition to normal plaque morphology, small pinprick plaques were occasionally observed on the triple-alanine mutant plates, which suggested the possibility that the triple-alanine insertion generated a temperature sensitive (ts) phenotype. Plating of several induction preparations of triple alanine phage and incubating at either 30 °C or 37 °C resulted in a roughly 3–4 log increase in titer when plated at the lower temperature, confirming the hypothesis that these phages were ts (Figure 6). The wild-type phage did not exhibit this trait, since titers remained consistent at both 30 °C and 37 °C. To further classify the ts triple-alanine mutant, we asked if the triple-alanine phages were stable at 37 °C. Phage stocks were split and incubated for 2 h at either room temperature or at 37 °C, then plated and titered at 30 °C. While the wild-type phage preparations maintained their stability and did not decrease the titer, the triple-alanine mutant decreased the titer greater than 1.2 logs when incubated at 37 °C (Figure 7).

## 4. Discussion

### 4.1. Stabilizing Effect of the A-Domain and Restriction of Scaffolding Protein Escape

The A-domain of the P22 coat protein was originally identified as a protease-sensitive site that undergoes substantial conformational rearrangement during expansion and maturation. Protease sensitivity experiments demonstrated that the region becomes protease inaccessible upon maturation [21]; hydrogen/deuterium exchange studies demonstrate that the A-domain becomes highly exchange protected upon expansion [8], and the entire lattice becomes more stable.

Thermodynamic studies indicate that while the expansion is exothermic [17], the activation energy required for expansion is quite high [22]. Strikingly, cleavage or tethering of the loop appears to lower the activation energy for expansion, suggesting that rearrangement of the loop poses an energetic barrier [8,21]. In this study, we found that the insertion of a glycine-flanked RGD sequence increased the activation energy required for expansion, adding to the evidence that the rearrangement of this loop is a barrier to maturation. However, the expanded RGD particles formed appear to be less stable, as it proved impossible to obtain the wiffle ball form.

The structure of the wild-type wiffle ball form has been analyzed by cryo-electron microscopy and three-dimensional image reconstruction [23] and has been found to be largely similar to the structure of the *in vivo* mature phage lattice, except absent pentamers [12]. The failure to obtain the wiffle ball for the RGD particles is presumably due to the fact that the interactions stabilizing the hexamer are perturbed by the insertions with the consequence that the entire lattice dissociates concurrently. In the mature lattice, the hexamers and the A-domain loop have become symmetrical, which closes the capsomere pores. In contrast to the wiffle ball form, no three-dimensional structure of the expanded form has been solved, despite having a similar diameter in negatively-stained micrographs [17]. Biochemical experiments in which procapsids were loaded with GFP and were heat expanded demonstrated that the expanded form retains the pentamers [11]. It is well documented that stress in the capsid is transmitted to the pentamers, which is presumed to play a role in penton dissociation during wiffle ball formation [24,25].

Some insights into the expansion pathway can be gained from studies with HK97 [26,27,28,29,30,31]. Despite sharing no sequence similarity, HK97 and P22 share a similar coat protein fold and arrangement of coat protein subunits within the lattice. HK97 undergoes a multi-step maturation process. In the transition between Prohead II to the first expansion intermediate, EI-1, the skew character of the hexons has been resolved, and presumably, all of the A-domain interactions have become equivalent though the spine helix and the P-domain remain in a strained conformation. This strain gives rise to an exothermic transition through the second expansion intermediate, EI-2, that drives the subsequent crosslinking to stabilize the capsid and completes the HK97 maturation process. The expanded form of P22 also occurs after an exothermic transition. This favors the notion that the expanded structure correlates with the HK97 expansion intermediate, EI-2, and therefore, the lack of stability in the RGD particles is a result of the inability to form stabilizing A-domain loop interactions. Comparing the alanine mutants, it is clear that insertions of up to two alanine residues maintains these stabilizing A-loop interactions, as is suggested by the near wild-type titers for these mutants. The drop in titer and temperature sensitivity for inductions of the triple-alanine mutant (T183AAA), as well as the absence of titer for the unflanked RGD demonstrate the structural limit to these stabilizing A-domain interactions.

*In vivo*, the sucrose gradient experiments suggest that the RGD insertions allow procapsid formation, but block DNA packaging. The GuHCl extraction studies suggest that release of the scaffolding protein is made more difficult by the insertions, perhaps because of steric effects. Mutational studies have shown that preventing scaffolding exit inhibits DNA packaging [32]. The steric occlusion model is consistent with the observation that the T183A and T183AA insertions are capable of producing phage, while the T183AAA is temperature sensitive for stability.

### 4.2. A-Domain Has Strong Selection Pressure toward Near-Wild-Type Sequences in Vivo

There are many phage with capsid proteins that adopt the HK-97-like fold, yet the sequence identity and overall structures of these capsids are heterogeneous [33]. This common fold implies that these phage have undergone strong selection pressure over time to adopt these conformations. Such strong selection pressure to maintain the HK-97-like fold suggests that the A-domain would only be permissive of one residue at the 183 codon and that any further additions to this region would ultimately result in reversion to a wild-type-like sequence length. This study demonstrates that both T183A and T183AA mutations are capable of producing phage, but any additional insertions result in large drops in titer. Since producing infectious phage is inherently a strong selection pressure, it was interesting to see that revertant phages from the induction of T183AAA and T183AAAA lysogens always resulted in double-alanine genotypes.

### 4.3. A-Domain Manipulations Can Produce Conditionally-Stable Procapsids

One of the main requirements of utilizing viruses as nanomedicine delivery devices is that the capsid must controllably dissociate to release drug [6]. The generation of a temperature-sensitive phenotype for the T183AAA mutant phage indicated that, in addition to interrogating maturation, the A-domain could be used to alter structural stability. Previous work has generated bone binding P22 procapsids that increase the charge localization on the surface of the immature structure by adding Glu residues at the A-domain loop [5]. The robust nature of assembly-permissive mutations at the A-domain loop coupled with the role this region plays in maturation allows for the design of capsids that are controllably and conditionally stable at certain temperatures, pH and other factors.

## Supplementary Files

Supplementary File 1 Supplementary Materials (PDF, 522 KB)

Supplementary File 2 Supplementary Movie (MP4, 8514 KB)
