# Supplementary material for: The Role of the Coat Protein A-Domain in P22 Bacteriophage Maturation
Source: Viruses. 2014 Jul 14;6(7):2708–22. doi: 10.3390/v6072708 (PMC4113789; doi:10.3390/v6072708)
Supplement: Supplementary File 1 — Supplementary Materials (PDF, 522 KB) [file viruses-06-02708-s001.pdf]

Supplementary Materials

The Role of the Coat Protein A-Domain in P22 Bacteriophage Maturation

David S. Morris and Peter E. Prevelige Jr.

Figure S1. Amplicon size assay. (**A**) Primers were designed to confirm the proper positioning of the TetRA cassette into the P22 coat protein gene. (**B**) PCR amplification using the primers designed confirmed the proper placement of the selection marker into the coat protein sequence.

Amplicon Size Primers	Sequence (5' to 3')
P22 check FOR	CGC AAG CTG GCG AAC AAC GTT G
P22 check REV	GTT TCG GAG AGC GCA GGA CAT C
TetRAnew FOR	CCC CAC AGC GCT GAG TGC AT
TetRAnew REV	ACT GGG CGC CGA CCA AAT CG
TetRP22FOR	GCA GAC GCC TGG AAC TTT GTG GCC GAC GCA GAA GAA ATC ATG TTC TTA AGA CCC ACT TTC ACA TT
TetRAP22REV	CTG AAT GGT GCC ATC TCG GTA TGC TTC TTC AGG AAT ACG CCC GAA CTA AGC ACT TGT CTC CTG
TetAR-Red-CheckFOR*	GAT CAA GAG CAT CAA GTC GC
TetAR-Red-CheckREV*	TCA GCA AGG TGC TTT ACA GG

(A)

Primer Sets	Expected (bp)
1. P22 check FOR + REV	2,143
2. TetRAnew FOR + REV	713
3. TetRP22FOR + TetRAP22REV	2,001
4. TetAR-Red-CheckFOR + P22check FOR	327
5. TetAR-checkREV + TetRP22REV	265

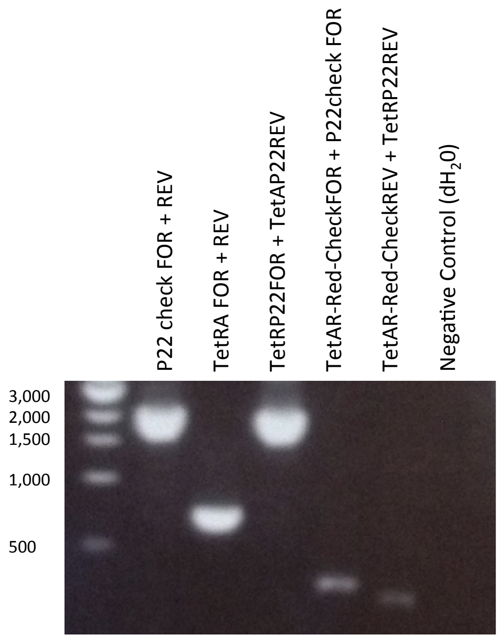

(B)

Table S1. Primers used for mutagenesis methods (**A**) Quickchange Primers. Standard Quickchange protocols were used to manipulate the coat protein sequence in the P22 assembler plasmid. (**B**) Oligo-extension primers. Oligo extension PCR was performed to generate the lambda-red homology sequences surrounding the designed manipulations. (**C**) Manipulation primers. Primers were used with oligo extension sets to generate mutations surrounded by the P22 coat protein homology for use in lambda-red recombineering.

Quickchange Primers	Sequence (5' to 3')
RGDforward	GAC TAC AAA AAA GCG GGT TAC GAC CTG GGA GGA GGA CGC GGT GAC GGA GGA GGA AAG CGT GAC ATC TTC GGG CGT ATT
RGDreverse	AAT ACG CCC GAA GAT GTC ACG CTT TCC TCC TCC GTC ACC GCG TCC TCC TCC CAG GTC GTA ACC CGC TTT TTT GTA GTC
P22switch	GAC ATG GGG ACA AGC TAT TTT TTT AAC CCT CAG GAC
P22switchREV	GTC CTG AGG GTT AAA AAA ATA GCT TGT CCC CAT GTC

(**A**)

Oligo Extension Primer	Sequence (5' to 3')
5218to5277for	ACT AAT ACC GCA GAC GCC TGG AAC TTT GTG GCC GAC GCA GAA GAA ATC ATG TTC TCC CGC
5260to5349for	GAA ATC ATG TTC TCC CGC GAA CTT AAC CGC GAC ATG GGG ACA TCG TAC TTC TTC AAC CCT CAG GAC TAC AAA AAA GCG GGT TAC GAC CTG
5353to5442rev	GCG CAG GAC ATC ATC GAA GCC AGC GAC CTG ACG CTG AAT GGT GCC ATC TCG GTA TGC TTC TTC AGG AAT ACG CCC GAA GAT GTC ACG CTT

(**B**)

Manipulation Primer	Sequence (5' to 3')
RGDrev (no linker)	CCC GAA GAT GTC ACG CTT GTC TCC CCT CAG GTC GTA ACC CGC TTT
G9rev	CCC GAA GAT GTC ACG CTT ACC TCC ACC TCC ACC TCC ACC TCC ACC CAG GTC GTA ACC CGC TTT
G3rev	CCC GAA GAT GTC ACG CTT TCC ACC TCC CAG GTC GTA ACC CGC TTT
T183A REV	CCC GAA GAT GTC ACG CTT CGC CAG GTC GTA ACC CGC TTT
T183A2 REV	CCC GAA GAT GTC ACG CTT CGC CGC CAG GTC GTA ACC CGC TTT
T183A3 REV	CCC GAA GAT GTC ACG CTT CGC CGC CGC CAG GTC GTA ACC CGC TTT
T183A4 REV	CCC GAA GAT GTC ACG CTT CGC CGC CGC CGC CAG GTC GTA ACC CGC TTT

(**C**)

Figure S2. Extraction of scaffolding from procapsids by guanidine hydrochloride. Scaffolding protein is retained more readily in the RGD procapsids than the wild-type. The data shown are an average of two experiments. The error bars shown are the standard error of the mean for each data point. The error bars are smaller than the data point icons in most cases.

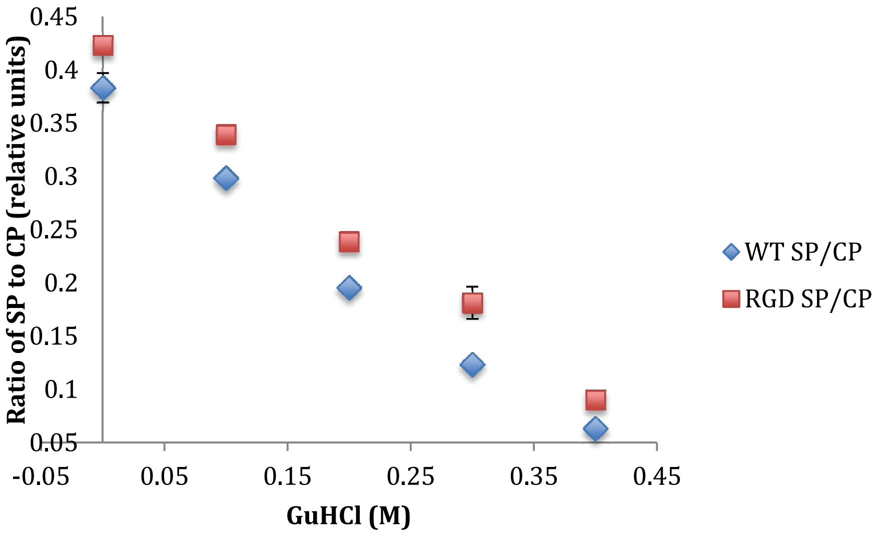

Figure S3. RGD strain titers to test sterics. Strains containing glycline flanked RGD, nine glycine residues, unflanked RGD, and three glycine residues were induced and titered. None of the mutant strains produced infectious particles.

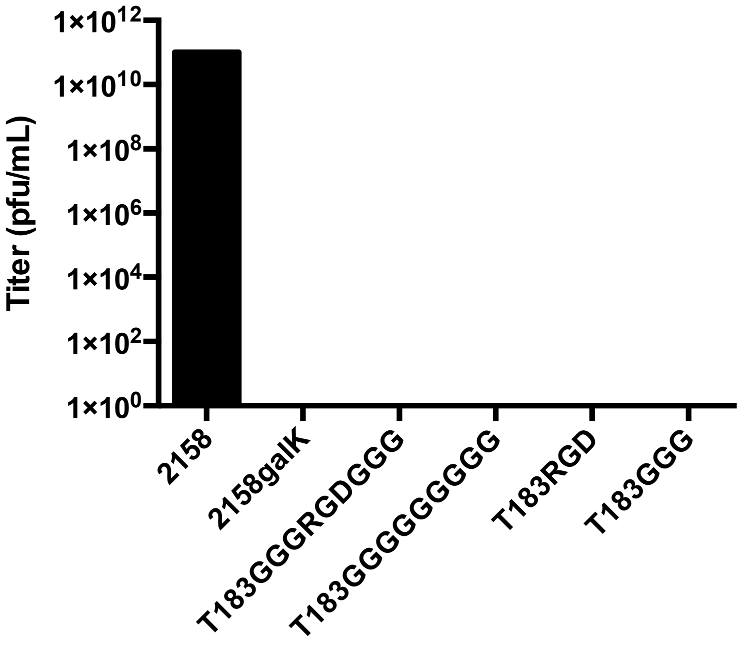

Movie 1. Transition between the immature (open) and mature (closed pore) form. Generated in Chimera (Pettersen, 2004) from PDB: 2XYY and 2XYZ (Chen, 2011).

File: maturationP22.mp4
